# Pre-service mathematics teachers' use of artificial intelligence: an extended technology acceptance model with self-efficacy (the case of ChatGPT)

**DOI:** 10.3389/fpsyg.2026.1826980

**Published:** 2026-05-28

**Authors:** Baris Demir

**Affiliations:** Department of Mathematics Education, Faculty of Education, Kocaeli University, Kocaeli, Türkiye

**Keywords:** artificial intelligence, ChatGPT, pre-service mathematics teachers, self-efficacy, technology acceptance model

## Abstract

**Introduction:**

This study examines pre-service mathematics teachers' use of ChatGPT in instructional processes within the framework of the Technology Acceptance Model (TAM), extended with pedagogical AI self-efficacy. The study investigates the relationships among perceived ease of use, perceived usefulness, attitudes toward use, actual use, and self-efficacy regarding the use of ChatGPT in mathematics teaching.

**Methods:**

The study was conducted with 167 pre-service mathematics teachers. Data were analyzed using Partial Least Squares Structural Equation Modeling (PLS-SEM).

**Results:**

The findings revealed that perceived ease of use significantly predicted perceived usefulness (β = 0.648, *p* < 0.001) and directly influenced actual use (β = 0.311, *p* < 0.001). Perceived usefulness significantly predicted attitudes toward use (β = 0.638, *p* < 0.001), while attitudes toward use significantly affected actual use (β = 0.399, *p* < 0.001). In addition, pedagogical AI self-efficacy strongly predicted perceived ease of use (β = 0.647, *p* < 0.001) and indirectly contributed to technology acceptance.

**Discussion:**

The findings confirm the explanatory power of TAM in the context of generative AI tools and highlight the critical role of self-efficacy in shaping pre-service teachers' acceptance of ChatGPT. The study provides important implications for the integration of AI tools into mathematics teacher education and supports the pedagogically grounded use of generative AI in teaching practices.

## Introduction

Artificial intelligence (AI) technologies have become indispensable in many domains and are increasingly being integrated into education. These developments aim to enhance educational systems through the use of AI applications.

Human responses to new technologies have long attracted scholarly attention (Yorulmaz and Alnipak, 2020). Research conducted during this process has generally been based on sociological and psychological theories. The Technology Acceptance Model (TAM) addressed in this research dates back to the reasoned action theory proposed by Fishbein and Ajzen in 1975 (Davis, 1989). According to this approach, which argues that human behavior always arises for specific reasons and is shaped by the reactions they receive from their environment, the perceived behavioral control variable, based on the assumption that human actions influence their own behavior and intentions, affects the behavior in question (Ajzen, 1991).

Recent studies on ChatGPT have generally focused on its effects on business, economics, security, health, education, and other sectors (Biswas, 2023; Aktay et al., 2023). Particularly in the education sector, the potential of such artificial intelligence applications is increasingly being understood (Rahman and Watanobe, 2023). In educational contexts, ChatGPT can be used by both students and educators. ChatGPT can respond to questions immediately and provide content on a wide range of topics. It supports both students and educators by facilitating content generation, lesson preparation, and instructional design. Similarly, students can also receive support from ChatGPT in solving complex problems and questions, writing essays, and accelerating their learning by explaining a topic (Kasneci et al., 2023).

Given these capabilities, ChatGPT represents one of the most visible examples of generative artificial intelligence (GenAI) in educational contexts. Its ability to support explanation, feedback, and problem- solving processes through natural language interaction suggests that such tools may play an important role in future teaching and learning environments (Kasneci et al., 2023). Therefore, examining pre-service mathematics teachers' use of ChatGPT is essential for understanding the integration of GenAI tools into mathematics education.

Although recent studies have highlighted the potential of ChatGPT in educational contexts, empirical research examining pre-service mathematics teachers' acceptance of ChatGPT within the framework of the TAM remains limited. However, the availability of AI tools such as ChatGPT does not automatically lead to their effective adoption in teaching processes. Research indicates that pre-service teachers may experience difficulties in using such tools in pedagogically meaningful ways, and their adoption decisions can be influenced by factors such as AI literacy, pedagogical preparedness, self-efficacy, and trust (Kasneci et al., 2023; Cotton et al., 2023). In addition, prior research emphasizes a gap between perceived usefulness of technology and its actual classroom integration (Ertmer and Ottenbreit-Leftwich, 2010). These challenges highlight the need to examine the factors influencing pre-service teachers' acceptance and use of ChatGPT in mathematics teaching.

The aim of this study is to examine pre-service mathematics teachers' use of ChatGPT, an AI application that has been increasingly used in education as technology has developed. From the TAM perspective, the perceived ease of use (PEOU), perceived usefulness (PU), attitudes toward use (ATU), actual use (AU), and self-efficacy (SE) of pre-service mathematics teachers regarding the use of ChatGPT in education constitute the focus of our study.

This study makes several important contributions to the literature. First, it extends the TAM by incorporating pedagogical AI self-efficacy as a key antecedent in the context of generative AI tools. Second, it provides empirical evidence of pre-service mathematics teachers' acceptance and use of ChatGPT, a topic that remains underexplored in mathematics education. Finally, the study offers practical insights into the factors that may facilitate or hinder the integration of AI tools into teaching processes.

In addition, self-efficacy emerges as a critical factor in understanding pre-service teachers' use of AI-based tools. Pedagogical AI self-efficacy refers to teachers' beliefs in their capability to integrate AI technologies effectively into teaching processes. This competence is particularly important, as it influences teachers' confidence in using AI tools and their willingness to adopt these technologies in instructional practices. Within the TAM, self-efficacy is considered an important antecedent that shapes perceived ease of use and perceived usefulness, thereby indirectly influencing technology acceptance and usage behavior (Bandura, 1997; Teo, 2011; Scherer et al., 2019).

This research aims to address the identified gap by posing the following research question.

**RQ**: How do pre-service mathematics teachers' perceptions and beliefs about ChatGPT and their self- efficacy influence their actual use of ChatGPT in mathematics teaching within the framework of the TAM?

## Literature review and research hypotheses

### Artificial intelligence and generative artificial intelligence in education

AI is considered one of the technologies that have led to significant transformations in education in recent years. AI systems can perform many functions, such as personalizing learning, providing instant feedback to students, generating learning analytics, and supporting teaching. For this reason, AI is considered a new paradigm in the development of educational technologies (Holmes et al., 2019). AI applications used in education include intelligent teaching systems, adaptive learning platforms, automatic assessment tools, and learning analytics systems (Luckin et al., 2016; Roll and Wylie, 2016; Zawacki-Richter et al., 2019; Holmes et al., 2022; Baker and Inventado, 2014).

Research on AI in education has expanded significantly in recent years (Luckin et al., 2016; Roll and Wylie, 2016; Chassignol et al., 2018; Zawacki-Richter et al., 2019; Baker and Smith, 2019; Chen et al., 2020; Ouyang and Jiao, 2021; Holmes et al., 2022; Kasneci et al., 2023). These studies show that AI technologies support learning processes, enhance instructional materials, and enable the development of personalized learning environments. A comprehensive systematic review conducted by Zawacki-Richter et al. (2019) shows that AI applications are rapidly becoming widespread, particularly in the context of higher education. Similarly, Holmes et al. (2022) state that artificial intelligence technologies can help teachers plan instruction more effectively and make students' learning experiences more interactive.

Recent developments in AI, particularly the emergence of GenAI technologies, have taken on a new dimension. GenAI systems can generate human-like text, provide contextual responses, and explain complex concepts (Brown et al., 2020; Bommasani et al., 2021; Kasneci et al., 2023). Thanks to advances in natural language processing technologies, such systems are considered to be new-generation digital tools capable of generating information and providing explanations (Dwivedi et al., 2023). Among these, ChatGPT has become one of the most widely used GenAI tools, capable of interacting with users and generating explanations across various fields. Research on the potential of GenAI tools such as ChatGPT in the field of education has also increased rapidly in recent years (Kasneci et al., 2023; Baidoo-anu and Owusu Ansah, 2023; Cotton et al., 2023; Lo, 2023; Rudolph et al., 2023; Tlili et al., 2024; Rahman and Watanobe, 2023).

Kasneci and colleagues (2023) state that ChatGPT can provide explanations that help students understand complex concepts and is an important tool that can support learning processes. Similarly, Cotton et al. (2023) state that GenAI tools can facilitate students' access to information and play a supportive role in teaching processes. Baidoo-anu and Owusu Ansah (2023) emphasize that ChatGPT offers significant opportunities in areas such as producing teaching materials, creating conceptual explanations, and supporting learning processes.

The potential of GenAI tools is particularly noteworthy in mathematics education. The mathematics learning process often requires understanding abstract concepts, developing problem-solving strategies, and using different forms of representation. In this context, AI tools that generate explanations and support problem-solving can significantly facilitate mathematics learning. AI-based tools can help students understand mathematical concepts and support learning processes by offering different solution strategies (Zawacki-Richter et al., 2019).

The effective use of AI technologies in educational settings is not solely dependent on their technical features. Research shows that the integration of educational technologies into teaching processes is largely related to pre-service teachers‘ perceptions, beliefs, and usage tendencies toward these technologies (Ertmer and Ottenbreit-Leftwich, 2010; Scherer et al., 2019). Pre-service teachers' perceptions of a technology as useful and easy to use can significantly influence their intention to use that technology in teaching processes and their usage behavior (Davis, 1989; Venkatesh and Davis, 2000; Teo, 2011). Therefore, theoretical models explaining the adoption of educational technologies provide an important theoretical framework for understanding how pre-service teachers evaluate new technologies and make decisions about using them (King and He, 2006; Venkatesh, 2000).

However, the existing literature indicates that empirical studies examining teachers‘ adoption of GenAI tools, such as ChatGPT, are limited. In particular, few studies have examined teachers' or pre-service teachers‘ use of ChatGPT within the framework of technology acceptance models in mathematics education. This highlights the need for theoretical and empirical research to explain the integration of GenAI tools into teaching processes. Therefore, examining the factors that influence pre-service mathematics teachers' adoption of GenAI tools such as ChatGPT is important for understanding the integration of AI technologies into educational environments. In this context, theoretical models of technology acceptance provide an important basis for understanding how pre-service teachers evaluate such technologies and which factors influence technology use.

### The use of ChatGPT in mathematics education

In recent years, research into the use of ChatGPT and similar GenAI tools in education has increased rapidly (Baidoo-anu and Owusu Ansah, 2023; Cotton et al., 2023; Kasneci et al., 2023; Lo, 2023; Rahman and Watanobe, 2023; Rudolph et al., 2023; Tlili et al., 2024). These studies show that systems based on large language models offer significant opportunities to provide explanatory answers to students‘ questions, to generate content that supports learning and to facilitate students' problem-solving.

Research on the use of ChatGPT in educational settings reveals that this technology can serve different functions in students‘ learning processes. In particular, ChatGPT's ability to generate explanations of complex concepts, offer alternative solution strategies, and guide students in their problem-solving processes increases this tool's potential to support learning processes (Kasneci et al., 2023; Rudolph et al., 2023). Furthermore, it is stated that ChatGPT facilitates students' access to information and can contribute to the creation of interactive learning environments in learning processes (Cotton et al., 2023; Baidoo-anu and Owusu Ansah, 2023).

In the context of mathematics education, AI-supported tools show significant potential, particularly for supporting problem solving. Mathematics learning processes generally require understanding abstract concepts and developing different solution strategies. Therefore, AI tools that can generate explanations and support problem-solving processes can provide significant support in mathematics learning processes (Holmes et al., 2022; Kasneci et al., 2023). Furthermore, it is stated that such tools can offer different solution paths to help students understand mathematical concepts and support personalized learning experiences (Rahman and Watanobe, 2023).

Teachers state that GenAI tools, such as ChatGPT, can be used for various purposes in instructional processes. Research shows that teachers can use such tools for various purposes, such as lesson planning, developing teaching materials, generating sample questions, and creating explanations that support students‘ conceptual understanding (Baidoo-anu and Owusu Ansah, 2023; Lo, 2023; Tlili et al., 2024). Furthermore, it is noted that systems based on large language models can be used as digital assistants in teachers' teaching processes and can contribute to teachers planning their teaching activities more efficiently (Rudolph et al., 2023; Rahman and Watanobe, 2023; Aydin and Karaarslan, 2023).

Previous studies on the use of technology in mathematics teaching show that teachers' use of digital tools can support students' mathematical learning processes (Niess, 2005; Goos, 2010; Pierce and Stacey, 2010; Li and Ma, 2010; Drijvers, 2013; Trouche, 2004; Artigue, 2002; Hoyles and Lagrange, 2010; Drijvers et al., 2010). These studies reveal that technology integration in mathematics teaching offers teachers' new pedagogical opportunities and can contribute to students' understanding of mathematical concepts through multiple representations. In particular, it is stated that digital tools can support teachers' problem-creation processes, help students see different solution strategies, and contribute to the visualization of mathematical concepts (Niess, 2005; Goos, 2010; Pierce and Stacey, 2010). In this context, GenAI tools, such as ChatGPT, could offer teachers new pedagogical opportunities in mathematics instruction.

However, a review of the literature on ChatGPT in education reveals that existing studies largely focus on students‘ perceptions of these technologies. Studies examining teachers' and pre-service teachers‘ perceptions of ChatGPT use and the factors influencing their use of this technology in teaching practice are limited. Few studies have examined trainee teachers' use of ChatGPT mathematics education within the framework of technology acceptance models. This situation makes it important to examine the factors that influence trainee teachers‘ use of ChatGPT. Therefore, it is important to apply theoretical models of technology acceptance to explain trainee teachers' perceptions of ChatGPT and their behavior when using this technology in teaching. The study's theoretical framework is based on the TAM.

### Technology acceptance model (TAM)

TAM is one of the most widely used models for explaining individuals' adoption and use of new technologies (Davis, 1989). The model suggests that individuals‘ intentions to use a technology and their usage behavior are largely shaped by their perceptions of that technology (Davis, 1989; Venkatesh and Davis, 2000). Initially developed in the field of information systems, this model has subsequently been applied across different fields and has become widely used, particularly in educational technology research (King and He, 2006; Scherer et al., 2019).

According to TAM, two fundamental perceptions play a decisive role in individuals‘ adoption of a technology: PU and PEOU (Davis, 1989; Venkatesh and Davis, 2000). PU refers to an individual's belief that using a particular technology will improve their performance, while PEOU refers to an individual's perception that using the technology in question will not require effort (Davis, 1989). These two perceptions are considered important variables in explaining individuals' attitudes toward technology and their technology usage behavior (Venkatesh et al., 2003; King and He, 2006).

The model also suggests that PEOU not only directly influences technology usage but can also affect the perception of the technology as beneficial (Davis, 1989; Venkatesh and Davis, 2000). Therefore, when individuals perceive a technology as easy to use, they may be inclined to think that the technology is more beneficial. Numerous studies in the literature show a significant relationship between PEOU and PU (Venkatesh et al., 2003; King and He, 2006; Sun and Zhang, 2006).

Although initially developed in the field of information systems, it has gradually become widely used in educational technology research. Studies conducted in the field of education indicate that the PU and PEOU variables play a significant role in explaining the technology usage behaviors of teachers and pre-service teachers (Teo, 2011; Scherer et al., 2019). It is stated that pre-service teachers' decisions to use new technologies in teaching processes are closely related to their perceptions of the contributions these technologies will make to teaching processes (Ertmer and Ottenbreit-Leftwich, 2010).

With the recent introduction of AI, including GenAI tools, in educational settings, technology acceptance models are being used to explain the adoption of these technologies. Research shows that individuals' intentions to use AI-based systems are significantly explained by variables such as perceived benefit, perceived ease of use, and attitudes toward technology (Chatterjee and Bhattacharjee, 2020; Schepman and Rodway, 2020; Dwivedi et al., 2023). Therefore, TAM provides a suitable theoretical framework for explaining the use of GenAI tools, such as ChatGPT, in educational settings.

However, the technology acceptance literature indicates that the factors influencing individuals‘ decisions to use new technologies are not limited to perceptual variables. In this context, it is stated that individuals' beliefs about their competence in using technology also play an important role in the technology acceptance process (Bandura, 1997; Compeau and Higgins, 1995; Teo, 2011). In this study, the Technology Acceptance Model was expanded to include self-efficacy. The self-efficacy variable is discussed in detail in the next section.

### Perceived usefulness

PU refers to an individual's belief that using a particular technology will improve their performance (Davis, 1989). According to TAM, perceived usefulness is considered one of the most important variables in explaining individuals' behavior in adopting and using new technologies (Venkatesh and Davis, 2000; Venkatesh et al., 2003). Research shows that when individuals believe a technology will help them perform their tasks more efficiently, their tendency to use that technology increases significantly (Gefen and Straub, 2000; Sun and Zhang, 2006).

Although the concept of PU was developed in the field of information systems, it has subsequently become widely used in educational technology research. Studies in the field of education show that teachers‘ and pre-service teachers' perceptions of technology use are largely related to their assessments of the contributions of these technologies to teaching processes (Legris et al., 2003; Marangunić and Granić, 2015). Pre-service teachers are more likely to adopt a technology when they perceive it as supportive of teaching processes, beneficial for student learning, and effective in enhancing instructional practices (Scherer et al., 2019).

With the recent introduction of AI and GenAI tools in educational settings, the perceived benefits of these technologies have become an important topic in research. Studies show that AI-based systems can provide pre-service teachers with various advantages, such as developing teaching materials, producing learning content, creating sample questions, and providing explanatory answers to students' questions (Kasneci et al., 2023; Tlili et al., 2024). It is stated that GenAI tools, such as ChatGPT, can make important contributions to pre-service teachers in terms of planning teaching processes, developing content, and supporting students' learning processes (Baidoo-anu and Owusu Ansah, 2023; Lo, 2023). In this context, pre-service teachers' perception that GenAI tools, such as ChatGPT, are useful for teaching is an important factor that may influence their attitudes toward these technologies and their tendency to use them.

### Perceived ease of use

PEOU refers to an individual's belief that using a particular technology will not require effort (Davis, 1989). Ease of use is considered an important determinant of technology adoption. Perceiving a technology as complex, difficult to understand, or time-consuming to use may reduce users' likelihood of adopting it (Agarwal and Prasad, 1999; Lee et al., 2003). Conversely it is stated that technologies with user-friendly interfaces that are easy to understand and accessible are adopted more quickly (Gefen et al., 2003).

PEOU not only directly affects technology use but also influences users' perceptions of the technology's usefulness. The perception that a technology is easy to use may lead individuals to believe that it will contribute more to the fulfillment of their tasks (Davis, 1989). Therefore, many studies have shown that perceived ease of use significantly affects perceived usefulness (Sun and Zhang, 2006; Marangunić and Granić, 2015).

Research conducted in the field of educational technology has also revealed similar findings. The ease of use of technologies plays an important role in teachers' and pre-service teachers' integration of digital tools into teaching processes. Technologies perceived as easy to use are preferred more in teaching environments (Inan and Lowther, 2010; Tondeur et al., 2017). Furthermore, it is stated that pre- service teachers' perception of the ease of use of technological tools supports the development of positive perceptions toward these tools (Scherer et al., 2019).

With the proliferation of GenAI tools in recent years, the ease of use of these systems has also become an important research topic. Since large language model-based systems enable interaction through natural language, they allow users to interact with these systems without requiring technical knowledge (Kasneci et al., 2023). This feature may contribute to the rapid adoption of GenAI tools such as ChatGPT by teachers and pre-service teachers (Baidoo-anu and Owusu Ansah, 2023; Lo, 2023). In this context, PEOU may influence the perceived usefulness of ChatGPT in mathematics teaching. Accordingly, the following hypothesis has been developed:

*H1: The perceived ease of use of ChatGPT in mathematics teaching positively influences its perceived usefulness*.

### Attitude toward use

ATU is defined as an important psychological variable that expresses an individual's positive or negative evaluations of using a particular technology and can influence technology usage behavior (Davis, 1989; Venkatesh et al., 2003). The concept of attitude is accepted as an important variable in explaining individuals‘ behavior in social psychology literature (Fishbein and Ajzen, 1975; Ajzen, 1991). According to this approach, individuals' tendencies to perform a specific behavior are largely shaped by their attitudes toward that behavior.

In the technology acceptance literature, attitude is also considered an important variable influencing users' technology-use behavior. Research shows that individuals' perception of a technology as useful and easy to use positively influences their attitude toward that technology (Davis, 1989; Venkatesh et al., 2012). Developing positive attitudes toward new technologies can increase users' likelihood of adopting and using these technologies (Kim and Kankanhalli, 2009).

Studies on educational technologies show that teachers' and pre-service teachers' attitudes toward technology play an important role in their technology use. Prospective teachers' perceptions that technology contributes to the teaching process and their belief that technology is easy to use can support the development of positive attitudes toward these tools (Ertmer and Ottenbreit-Leftwich, 2010; Teo, 2011).

The proliferation of AI and GenAI technologies in recent years has made individuals‘ attitudes toward these technologies an important research topic. Studies show that users' attitudes toward AI systems can significantly influence their intentions to use these technologies and their usage behavior (Chatterjee and Bhattacharjee, 2020; Schepman and Rodway, 2020). Furthermore, it is stated that the perceived usefulness and ease of use of AI technologies can shape individuals‘ attitudes toward these systems (Shin, 2021; Dwivedi et al., 2023). In this context, the perceived usefulness and ease of use of GenAI tools, such as ChatGPT, in mathematics education are important factors influencing pre-service teachers' attitudes toward these technologies. Accordingly, the following hypotheses have been developed:

*H2: The perceived usefulness of ChatGPT in mathematics teaching positively influences pre-service teachers' attitudes toward ChatGPT*.

*H3: The perceived ease of use of ChatGPT in mathematics teaching positively influences pre-service teachers' attitudes toward ChatGPT*.

### Actual use

AU is a variable that expresses individuals‘ actual usage behavior of a particular technology and is considered one of the most important outputs of the technology acceptance process (Davis, 1989). According to the TAM, individuals' attitudes toward using a technology can significantly influence their use of that technology. Developing positive attitudes toward a technology can increase the likelihood of users adopting it (Davis, 1989; Venkatesh et al., 2003).

Subsequent studies examining ATU also show that individuals' attitudes and perceptions of technology are important factors influencing technology use. In particular, the Unified Theory of Acceptance and Use of Technology (UTAUT) reveals that individuals' technology usage behavior is influenced by various perceptual and psychological factors (Venkatesh et al., 2003, 2012). These studies show that individuals' perceptions of technology as useful and their beliefs that technology is easy to use can directly influence technology use.

Research conducted in the field of educational technology also reveals that the technology-use behavior of teachers and pre-service teachers is largely related to their perceptions and attitudes toward these technologies. Pre-service teachers' belief that technology contributes to the teaching process and their perception that this technology is easy to use may increase the likelihood of these tools being used in teaching environments (Inan and Lowther, 2010; Tondeur et al., 2017).

Recent studies on the use of AI-based systems in educational settings have also yielded similar findings. Research indicates that users' perceptions of the perceived benefits and ease of use of AI technologies can significantly influence their behavior in using these systems (Chatterjee and Bhattacharjee, 2020; Dwivedi et al., 2023). The use of GenAI tools, such as ChatGPT, in teaching may similarly be shaped by users' perceptions and attitudes toward these tools. In this context, it is predicted that pre-service teachers' attitudes toward ChatGPT and their perceived usefulness of, and ease of use regarding, it may influence the use of ChatGPT in mathematics teaching. Accordingly, the following hypotheses have been developed:

*H4: Pre-service teachers' attitudes toward ChatGPT positively influence the use of ChatGPT in mathematics teaching*.

*H5: The perceived usefulness of ChatGPT positively influences its use in mathematics teaching*.

*H6: The perceived ease of use of ChatGPT in mathematics teaching positively influences ChatGPT use*.

The literature indicates that variables such as PU and PEOU may also indirectly affect AU. In this context, it is stated that the attitude variable may play a mediating role in the relationship between perceived usefulness, perceived ease of use, and technology actual use (Davis, 1989; Venkatesh et al., 2003). Accordingly, the following hypotheses have been developed:

*H5a: The perceived usefulness of ChatGPT in mathematics teaching positively and indirectly influences its use through pre-service teachers' attitudes*.

*H6a: The perceived ease of use of ChatGPT in mathematics teaching indirectly and positively influences its use through pre-service teachers' attitudes*.

### Self-efficacy in technology use

Self-efficacy refers to individuals‘ beliefs about their capacity to successfully perform a specific task (Bandura, 1997). According to social cognitive theory, individuals' perceptions of self-efficacy can significantly influence their motivation, effort, and performance in carrying out a specific behavior. When individuals believe they can successfully perform a specific task, they tend to exert more effort toward that task and persist in their behavior despite the difficulties they encounter.

In the context of educational technologies, self-efficacy is an important variable in explaining teachers' and pre-service teachers' confidence and perceived competence in using digital tools. Research shows that perceptions of self-efficacy regarding technology can significantly influence the integration of technology into teaching processes (Compeau and Higgins, 1995; Venkatesh, 2000; Agarwal et al., 2000). In particular, pre-service teachers' high perceived competence in using digital tools may increase the likelihood of integrating these tools into teaching practice.

Studies conducted in the field of educational technology show that perceptions of self-efficacy regarding technology are significantly related to perceived ease of use and perceived usefulness, which are the core components of TAM. In particular, it has been found that self-efficacy directly influences individuals' perceptions of how easily they can use a technology and that this perception is linked to their assessments of the technology's usefulness (Teo, 2011; Scherer et al., 2019). Research conducted with samples of teachers and pre-service teachers similarly reports that beliefs about technological competence shape technology acceptance through PEOU and PU (Hsu and Ching, 2013; Tondeur et al., 2017).

The integration of digital technologies into teaching processes in the context of mathematics education is also closely related to pre-service teachers‘ self-efficacy beliefs regarding their ability to use these technologies for pedagogical purposes. Research shows that mathematics teachers' and pre-service teachers' perceptions of self-efficacy in using technology significantly influence the integration of technology in mathematics teaching (Niess, 2005; Goos, 2010; Pierce and Stacey, 2010). In particular, the use of digital tools in mathematics teaching can help teachers explain mathematical concepts using different representations, support problem-solving processes, and develop students‘ mathematical thinking skills.

In recent years, the number of studies on the use of GenAI tools in educational settings has increased. Research shows that large language model-based systems offer significant opportunities in terms of generating mathematical explanations, presenting alternative solution strategies, and supporting the development of teaching materials (Kasneci et al., 2023; Lo, 2023). However, the effective use of such tools in teaching practices is closely related to pre-service teachers' self-efficacy regarding the pedagogical use of these technologies.

In this study, self-efficacy is conceptualized not merely as technological competence grounded in technical skills but as pedagogical AI self-efficacy, which encompasses beliefs about the pedagogical, ethical, and critical use of AI tools. In this context, ChatGPT self-efficacy refers to pre-service teachers' beliefs about their ability to use ChatGPT effectively in mathematics teaching.

In the context of mathematics teaching, ChatGPT self-efficacy refers to pre-service teachers' beliefs about their competence in using ChatGPT appropriately in pedagogical contexts to explain mathematical concepts, generate alternative solution strategies, support problem-creation processes, and develop students‘ mathematical thinking. It also includes their awareness of the limitations of AI tools and their ability to use these tools critically. From this perspective, pre-service teachers with higher ChatGPT self-efficacy are expected to perceive the tool as easier to use and more useful in mathematics teaching.

The following hypotheses are proposed:

*H7: ChatGPT self-efficacy positively affects the perceived usefulness of ChatGPT in mathematics teaching*.

*H8: ChatGPT self-efficacy positively affects the perceived ease of use of ChatGPT in mathematics teaching*.

**Figure 1:**
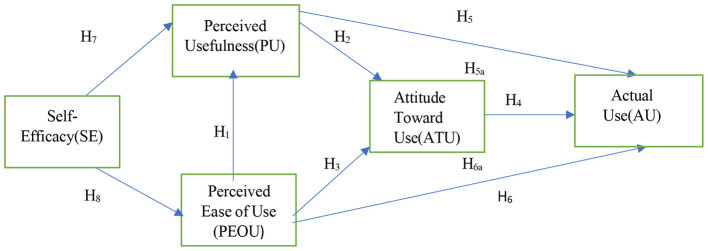
Conceptual model of ChatGPT acceptance in mathematics teaching based on an extended TAM.

The literature presented above reveals the key factors influencing the adoption of new technologies in an educational context. Drawing on the TAM and incorporating the self-efficacy variable into the model, this study proposes a conceptual model that explains pre-service mathematics teachers' use of ChatGPT in teaching mathematics. The proposed model assumes that PU, PEOU, and ATU affect the AU of ChatGPT for mathematics teaching, while SE affects PU and PEOU. In line with these theoretical relationships, the conceptual model of the study is presented in Figure 1.

## Methods

### Research design

This study employed a cross-sectional, quantitative survey design to examine the relationships among the variables in the proposed research model. The study was grounded in TAM and aimed to examine pre-service teachers' perceptions of the use of ChatGPT. In this context, the relationships among the PU, PEOU, ATU, AU, and SE variables were examined.

### Participants

Fourth-year pre-service mathematics teachers enrolled in teacher education programs at four universities in the Marmara Region of Türkiye, one of the country's most populous regions, participated in the study. A total of 167 pre-service teachers were included in the study, of whom 123 were female (73.7%) and 44 were male (26.3%). Fourth-year pre-service teachers were chosen for this study because these students actively participate in mathematics lessons in secondary schools as part of their teaching practice and thereby gain experience with teaching processes in real classroom settings. An appropriate sampling method was used to reach the participants, and participation in the study was voluntary. A total of 185 pre-service teachers were reached during the data collection process. Two control questions were added to the questionnaire to improve data quality in online surveys. Eighteen questionnaires that either lacked correct answers to the control questions or contained incomplete data were excluded from the dataset. Thus, the analyses were conducted using data obtained from 167 valid participants.

### Instruments

The measurement tool used in this study was developed based on TAM. The scale items were adapted from TAM scales commonly used in the literature, and the statements were adjusted to suit the context of ChatGPT's use in teaching mathematics. The literature indicates that a common approach to adapting TAM items for different technological systems is to modify the general “system” statements within scale items to reflect the relevant technological context. In this study, the item statements were also adapted to reflect the use of ChatGPT in mathematics teaching.

During the scale adaptation process, the items used in TAM studies were examined and reorganized to suit the context of the research. During the adaptation process, academics specializing in mathematics education and educational technologies were asked to evaluate the content validity of the items. Necessary adjustments were made to the wording of the items based on expert opinion. In addition, a pilot study was conducted with a limited number of pre-service teachers to assess the scale's comprehensibility and applicability. Based on feedback from the pilot study, minor linguistic adjustments were made to some items, and the final version of the scale was established. Some items, particularly within the ATU construct, showed conceptual overlap with perceived usefulness; however, rather than reallocating these items, they were instead removed based on the results of factor analysis to preserve construct clarity and discriminant validity.

The developed measurement tool consists of five sub-dimensions:

PU: Measures perceptions ChatGPT's contributions to mathematics teaching. PEOU determines the degree to which pre-service teachers perceive the use of ChatGPT as easy.

ATU: Measures pre-service teachers' positive or negative attitudes toward the use of ChatGPT. This study assesses the extent to which pre-service teachers use ChatGPT in mathematics teaching.

SE: Measures pre-service teachers' self-efficacy beliefs regarding their ability to use ChatGPT effectively in mathematics teaching.

The scale contains 29 items. The items are rated on a 5-point Likert scale (1 = Strongly Disagree, 5 = Strongly Agree). Some items in the scale are reverse-coded (R) to balance possible response tendencies. The questionnaire was administered online, and participants were informed that their participation in the study was voluntary and their responses would be evaluated anonymously. The items comprising the scale's subscales are presented below.

#### PU

Items adapted from and based on: Davis (1989); Venkatesh (2000); Lai and Li (2005); Abdaljaleel et al. (2024); Shaengchart et al. (2023) and Yilmaz et al. (2023).

PU1. Using ChatGPT in mathematics lessons makes my teaching tasks easier.

PU2. Using ChatGPT in mathematics lessons increases student engagement.

PU3. Using ChatGPT in mathematics lessons improves lesson efficiency.

PU4. Using ChatGPT in mathematics lessons improves my teaching performance.

PU5. Using ChatGPT in mathematics lessons helps complete instruction more quickly.

#### PEOU

Items adapted from and based on: Davis (1989); Venkatesh (2000); Premkumar and Bhattacherjee (2008); Lai and Li (2005); Strzelecki (2024); Abdaljaleel et al. (2024); Huang et al. (2017); Shaengchart et al. (2023) and Yilmaz et al. (2023).

PEOU1. Using ChatGPT in mathematics lessons is easy for me.

PEOU2. Learning to use ChatGPT in mathematics lessons is easy for me.

PEOU3. I think ChatGPT tools are easily accessible for mathematics lessons.

PEOU4. The use of ChatGPT in mathematics teaching is evident.

#### ATU

Items adapted from and based on: Davis (1989); Venkatesh (2000); Lai and Li (2005); Yilmaz et al. (2023) and Shaengchart et al. (2023).

ATU1. Using ChatGPT in mathematics lessons makes me happy.

ATU2. Using ChatGPT makes mathematics lessons more engaging.

ATU3. The use of ChatGPT in mathematics instruction makes teaching more difficult (R).

ATU4: I consider ChatGPT is important for the teaching of mathematics.

ATU5. Using ChatGPT in mathematics lessons may be beneficial.

ATU6. I believe that spending excessive time using ChatGPT during mathematics lessons is unnecessary (R).

ATU7. I feel the need to use ChatGPT in mathematics teaching.

ATU8. I enjoy teaching mathematics using ChatGPT.

ATU9. I find the use of ChatGPT in mathematics lessons unnecessary (R).

#### AU

Items adapted from and based on: Davis (1989); Lai and Li (2005); Shaengchart et al. (2023); Strzelecki (2024) and Yilmaz et al. (2023).

AU1. I encourage students to use ChatGPT to support self-directed learning during mathematics lessons.

AU2. I use ChatGPT in mathematics lessons.

AU3. I avoid using ChatGPT in mathematics lessons because it is too time-consuming (R).

AU4. I use ChatGPT frequently during mathematics lessons.

AU5. I do not use ChatGPT in mathematics lessons (R).

#### SE

Items adapted from and based on: Venkatesh and Davis (1996); Venkatesh (2000); Al-Haderi (2013); Teo (2011) and Abdaljaleel et al. (2024).

SE1. I am aware of the disadvantages of using ChatGPT during the planning and implementation of mathematics lessons.

SE2. I can take ChatGPT's limitations into account while teaching mathematics.

SE3. I have the knowledge and skills necessary to use ChatGPT in mathematics lessons.

SE4. I can inform students about the risks, ethical issues, and limitations of ChatGPT.

SE5. I feel confident using ChatGPT to teach mathematics.

SE6. When I encounter a problem while using ChatGPT, I can solve it.

The reliability and validity of the scale were assessed within the measurement model, and these findings are reported in detail in the Results section.

### Procedure

Data were collected via an online survey. Prior to data collection, a briefing was provided to ensure that participants had a basic awareness of the use of ChatGPT and AI technologies in education. In this context, pre-service teachers were shown a 15-minute informational video covering the basic features of ChatGPT, its working principles, and examples of its use in educational settings. The use of video- based stimuli in educational research is a common method that helps participants develop a shared context about the research topic and better evaluate the situation under investigation (Derry et al., 2010). After the participants viewed the video, the researchers explained the purpose of the research and emphasized to them that participation in the study was voluntary. Participants were also informed that their responses would be evaluated anonymously and used solely for scientific purposes. After the information session, participants were asked to complete an online questionnaire.

### Data analysis

The data obtained in the study were analyzed using IBM SPSS 26 and SmartPLS 4. The PLS- SEM method was preferred for testing the structural model in this study. PLS-SEM is widely used for exploratory models, small samples, and complex structural relationships (Hair et al., 2019, 2021). As the PLS-SEM method used in structural model analyses is an approach that is not sensitive to distribution assumptions, the normal distribution assumption was not considered a critical requirement (Hair et al., 2021). In the first stage, exploratory factor analysis (EFA) was applied to examine the factor structure of the dataset. In factor analysis, the scale's factor structure was evaluated using factor loadings, cross-loadings, and total variance explained. Items with low factor loadings were removed from the model, and the factor structure was re-examined. In the second stage, confirmatory factor analysis (CFA)was performed to evaluate the construct validity of the measurement model. At this stage, the factor loadings of the items were examined, and Cronbach's alpha and composite reliability (CR) were calculated to assess the internal consistency of the scales. Average Variance Extracted (AVE) values were examined to evaluate convergent validity. Furthermore, the Fornell–Larcker criterion, the Heterotrait–Monotrait ratio (HTMT), and cross-loadings were analyzed to assess the discriminant validity of the constructs. Additionally, the full-collinearity approach was used to assess potential common-method bias in the dataset by calculating variance inflation factors (VIFs). The relationships among the variables in the research model were tested using structural equation modeling (SEM). In the structural model analysis, path coefficients, explained variance (R^2^), and the model's direct and indirect effects were evaluated. Furthermore, the mediating relationships within the model were tested using bootstrapping.

## Results

### Testing measurement model

Cross-loadings were evaluated considering the specified threshold values, and the factor structure was adjusted where necessary. In the factor analysis literature, it is recommended that a reliable factor structure can be obtained if each factor consists of at least three items with factor loadings above 0.40 (Hair et al., 2010; Costello and Osborne, 2005; Tabachnick and Fidell, 2013). In line with these criteria, items with factor loadings below 0.40 were excluded from the model. In this context, one item from the PEOU dimension (PEOU3), six items from the ATU dimension (ATU1, ATU2, ATU4, ATU5, ATU6, and ATU9), one item from the AU dimension (AU5), and three items from the SE dimension (SE1, SE3, and SE5) were excluded from the analysis because their factor loadings were below 0.40.

The removal of more items from the ATU dimension than from other dimensions may be due to these items reflecting general attitudes toward ChatGPT use rather than adequately representing the attitude structure specific to mathematics teaching. This situation is frequently encountered in the literature, particularly when measuring new technologies within a specific disciplinary context. Items with low factor loadings were removed from the analysis to allow the measurement model to exhibit a more consistent, valid factor structure. After item removal, the analyses were repeated, revealing that the remaining items exhibited acceptable factor loadings.

Table 1 presents the results of the confirmatory factor analysis conducted to assess the measurement model. An examination of the standardized factor loadings indicates that all indicators load strongly on their respective latent constructs (Hair et al., 2021). The factor loadings for PU range between 0.736 and 0.834, while those for PEOU vary between 0.734 and 0.895. The items measuring SE exhibit factor loadings ranging from 0.742 to 0.810, whereas those for ATU range from 0.804 to 0.926. Finally, the indicators associated with AU demonstrate factor loadings between 0.719 and 0.881.

**Table 1:** Confirmatory factor analysis.

Items	PU	PEOU	SE	ATU	AU
PU1	0.829				
PU2	0.834				
PU3	0.736				
PU4	0.803				
PU5	0.765				
PEOU1		0.895			
PEOU2		0.734			
PEOU4		0.888			
SE2			0.788		
SE4			0.810		
SE6			0.742		
ATU3				0.926	
ATU7				0.841	
ATU8				0.804	
AU1					0.719
AU2					0.765
AU3					0.881
AU4					0.768

Table 2 presents result for internal consistency reliability and convergent validity of the measurement model, assessed using Cronbach's alpha (α), composite reliability (CR), and average variance extracted (AVE). The Cronbach's alpha values range from 0.679 to 0.853. Consistent with this perspective, the composite reliability (CR) values for all constructs range from 0.824 to 0.895, exceeding the recommended threshold of 0.70 and indicating satisfactory internal consistency across all latent variables. In addition, AVE values range from 0.609 to 0.737, all of which surpass the minimum criterion of 0.50, indicating that each construct explains more than half of the variance in its indicators. According to Hair et al. (2021), CR values above 0.70 and AVE values above 0.50 provide strong evidence of convergent validity.

**Table 2:** Alfa, CR, and AVE.

Variables	α	CR	AVE
PU	0.853	0.895	0.631
PEOU	0.798	0.879	0.710
SE	0.679	0.824	0.609
ATU	0.821	0.893	0.737
AU	0.790	0.865	0.617

Table 3 reports the results of the discriminant validity assessment based on the HTMT ratio. The HTMT criterion suggests that values below 0.90 indicate adequate discriminant validity between latent constructs, whereas values exceeding 0.90 may signal a lack of discriminant validity and potential conceptual overlap between constructs (Hair et al., 2021). As shown in Table 3, all HTMT values among the constructs are below the recommended threshold of 0.90.

**Table 3:** HTMT ratio.

Variables	PU	PEOU	SE	ATU	AU
PU					
PEOU	0.685				
SE	0.422	0.859			
ATU	0.853	0.608	0.494		
AU	0.804	0.753	0.580	0.867	

Table 4 presents the discriminant validity assessment of the measurement model based on the Fornell–Larcker criterion. According to this criterion, the square root of the average variance extracted (AVE) for each construct should be greater than its correlations with all other constructs, indicating that a latent variable shares more variance with its own indicators than with other latent variables (Fornell and Larcker, 1981). As shown in Table 4, the square roots of AVE values (reported on the diagonal in bold) for PU (0.794), PEOU (0.842), SE (0.781), ATU (0.859) and AU (0.785) are all higher than the corresponding inter-construct correlations presented in the off-diagonal elements of the matrix. These findings indicate that each construct demonstrates adequate discriminant validity, as none of the correlations between different constructs exceed the square root of the AVE of the related constructs. In line with the recommendations of Hair et al. (2021) the results provide empirical support that the constructs included in the model are conceptually distinct and that the measurement model satisfies the Fornell–Larcker criterion for discriminant validity.

**Table 4:** Fornell Larcker criterion.

Variables	PU	PEOU	SE	ATU	AU
PU	**0.794**				
PEOU	0.575	**0.842**			
SE	0.309	0.647	**0.781**		
ATU	0.722	0.520	0.365	**0.859**	
AU	0.661	0.629	0.424	0.701	**0.785**

### Common method bias

Common method bias was assessed using the full collinearity approach based on variance inflation factor (VIF) values. According to Kock (2015), VIF values below the threshold of 3.3 indicate that common method variance is unlikely to be a serious concern in PLS-SEM models. The results reveal that all VIF values (1.209 – 2.741) remain well below this critical threshold, suggesting that multicollinearity among the indicators is not problematic.

### Testing structural model

Prior to examining the hypothesized relationships within the structural model, the overall model quality and explanatory power were assessed using several model evaluation criteria recommended in the PLS-SEM literature. Specifically, model fit was evaluated using the standardized root mean square residual (SRMR), while the predictive accuracy of the endogenous constructs was examined using the coefficients of determination (R^2^ and adjusted R^2^). In addition, effect sizes were assessed using f^2^ values to determine the relative contribution of each exogenous construct to the explained variance of the endogenous variables.

Model fit was assessed using the standardized root mean square residual (SRMR). The SRMR for both the saturated and the estimated models was 0.103. Although SRMR values below 0.08 are often considered indicative of good model fit, prior research emphasizes that SRMR should be interpreted cautiously in the context of PLS-SEM. Unlike covariance-based SEM, PLS-SEM focuses on maximizing the explained variance of endogenous constructs rather than on reproducing the observed covariance matrix exactly. As a result, SRMR values in PLS-SEM applications may be relatively higher without necessarily indicating poor model fit (Henseler et al., 2016; Hair et al., 2022).

The explanatory power of the model was assessed using the coefficient of determination (R^2^). According to the guidelines proposed for PLS-SEM, R^2^ values of approximately 0.25, 0.50, and 0.75 can be interpreted as weak, moderate, and substantial explanatory power, respectively (Hair et al., 2022). The results indicate that the model explains 33.7% of the variance in PU, 41.8% of the variance in PEOU, 53.8% of the variance in ATU, and 60.4% of the variance in AU; comparable values were observed for adjusted R^2^. Based on these thresholds, the model's explanatory power can be considered moderate for PU and PEOU, and moderate to substantial for ATU and AU. These findings suggest that the proposed model demonstrates adequate predictive accuracy and effectively explains pre-service mathematics teachers' intentions and actual use of ChatGPT.

The effect sizes of the structural relationships were assessed using the f^2^ statistic to determine the relative contribution of each exogenous construct to the explained variance of the endogenous variables. According to Cohen (1988), f^2^ values of 0.02, 0.15, and 0.35 indicate small, medium, and large effect sizes, respectively. Based on these criteria, the effects of PEOU on SE (f^2^ = 0.719) and of PU on ATU (f^2^ = 0.580) can be classified as large, indicating substantial contributions to the explanatory power of the model. Medium effect sizes were observed for the impacts of PU on PEOU (f^2^ = 0.365) and for PEOU on AU (f^2^ = 0.156). In contrast, several paths exhibited small effect sizes (e.g., f^2^ = 0.010 and 0.036), suggesting a limited but non-negligible contribution to the explained variance.

Table 5 presents the direct relationships among the variables tested within the structural model. The findings show that PU has a positive and significant effect on ATU (β = 0.638; *t* = 7.711; *p* < 0.001). Similarly, the effect of PU on AU is positive and significant (β = 0.195; *t* = 2.343; *p* < 0.05). These results suggest that perceived usefulness plays a decisive role in shaping both ATU and AU. The effect of PEOU on PU is positive, strong, and statistically significant (β = 0.648; *t* = 10.845; *p* < 0.001). In addition, PEOU has direct and significant effects on both ATU (β = 0.150; *t* = 2.164; *p* < 0.05) and AU (β = 0.311; *t* = 5.380; *p* < 0.001). These findings indicate that ease of use influences usage behavior through both direct and indirect pathways. The effect of SE on PU is not statistically significant (β = −0.116; *t* = 1.410; *p* > 0.05). In contrast, SE has a very strong positive effect on PEOU (β = 0.647; *t* = 12.183; *p* < 0.001). This result suggests that individuals' perceptions of their own capabilities play an important role in their perceived ease of use of the system. Finally, ATU has a positive and statistically significant effect on AU (β = 0.399; *t* = 4.733; *p* < 0.001). This finding demonstrates that positive attitudes directly translate into actual use.

**Table 5:** Direct effects.

Relationships between variables	Std beta	Sample mean	Standard deviation	*T* statistics	*P* values
PU -> ATU	0.638	0.629	0.083	7.711	0.000
PU -> AU	0.195	0.195	0.083	2.343	0.019
PEOU -> PU	0.648	0.652	0.060	10.845	0.000
PEOU -> ATU	0.150	0.157	0.069	2.164	0.031
PEOU -> AU	0.311	0.310	0.058	5.380	0.000
SE -> PU	-0.116	−0.112	0.082	1.410	0.158
SE -> PEOU	0.647	0.649	0.053	12.183	0.000
ATU -> AU	0.399	0.398	0.084	4.733	0.000

Table 6 presents the indirect effects among the variables tested within the structural model. The findings reveal several mediating mechanisms that clarify how the constructs influence usage behavior, both partially and sequentially. First, PU has a positive and significant indirect effect on AU through ATU (β = 0.254; *t* = 3.837; *p* < 0.001). This result indicates that attitude plays a mediating role in the relationship between perceived usefulness and actual usage, suggesting that usefulness perceptions translate into usage primarily by fostering a positive attitude.

**Table 6:** Indirect effects.

Relationships between variables	Std. beta	Sample mean	Standard deviation	*T* statistics	*P* values
PU → ATU → AU	0.254	0.252	0.066	3.837	0.000
SE → PEOU → PU	0.419	0.423	0.057	7.388	0.000
PEOU → ATU → AU	0.060	0.063	0.032	1.882	0.060
PEOU → PU → ATU	0.414	0.412	0.074	5.558	0.000
SE → PEOU → ATU	0.097	0.103	0.047	2.057	0.040
PEOU → PU → AU	0.126	0.127	0.056	2.260	0.024
SE → PU → ATU	-0.074	−0.073	0.055	1.339	0.181
SE → PEOU → AU	0.201	0.201	0.042	4.846	0.000
SE → PU → AU	-0.023	−0.023	0.021	1.082	0.279
SE → PEOU → PU → AU	0.082	0.083	0.038	2.171	0.030
SE → PEOU → PU → ATU	0.267	0.267	0.054	4.997	0.000
SE → PEOU → ATU → AU	0.039	0.041	0.021	1.811	0.070
PEOU → PU → ATU → AU	0.165	0.165	0.049	3.390	0.001
SE → PU → ATU → AU	-0.029	−0.030	0.024	1.217	0.224
SE → PEOU → PU → ATU → AU	0.107	0.107	0.033	3.261	0.001

SE exerts a strong and significant indirect effect on PU through PEOU (β = 0.419; *t* = 7.388; *p* < 0.001). This finding highlights perceived ease of use as a key mediating mechanism through which individuals' self-efficacy enhances their perceptions of usefulness. Similarly, perceived ease of use has a significant indirect effect on attitudes through perceived usefulness (β = 0.414; *t* = 5.558; *p* < 0.001), indicating that ease of use shapes attitudes primarily by increasing perceived usefulness.

The indirect effect of PEOU on AU through ATU alone is marginal and not statistically significant at the 0.05 level (β = 0.060; *t* = 1.882; *p* = 0.060). However, PEOU significantly affects AU through PU (β = 0.126; *t* = 2.260; *p* < 0.05), indicating that PU plays a critical mediating role in the effect of perceptions of ease of use on AU. Regarding SE, its indirect effect on ATU through PEOU is positive and statistically significant (β = 0.097; *t* = 2.057; *p* < 0.05), reinforcing the role of ease of use as a mediator between individual capability beliefs and attitudinal responses. In contrast, the indirect effect of SE on ATU via PU is not significant (β = −0.074; *t* = 1.339; *p* > 0.05), suggesting that SE does not influence attitudes through usefulness alone. Several multi-step mediation paths yield significant results. SE has a positive and significant indirect effect on AU through PEOU(β = 0.201; *t* = 4.846; *p* < 0.001), whereas the indirect path through perceived usefulness alone is not significant (β = −0.023; *t* = 1.082; *p* > 0.05).Moreover, the sequential mediation path SE → PEOU → PU → AU is statistically significant (β = 0.082; *t* = 2.171; *p* < 0.05), indicating that self-efficacy influences usage behavior through a chain of perceptions of ease of use and usefulness. Similarly, SE has a significant indirect effect on ATU through the sequential path SE → PEOU → PU → ATU (β = 0.267; *t* = 4.997; *p* < 0.001).The extended mediation path PEOU → PU → ATU → AU is also significant (β = 0.165; *t* = 3.390; *p* < 0.01), emphasizing the cumulative role of usefulness and attitude in linking ease of use to actual usage behavior. In contrast, the indirect effects of SE on AU through ATU alone (β = −0.029; *t* = 1.217; *p* > 0.05) and through the longer chain SE → PEOU → ATU → AU (β = 0.039; *t* = 1.811; *p* > 0.05) are not statistically significant. Finally, the most comprehensive sequential mediation path, SE → PEOU → PU → ATU → AU, is positive and significant (β = 0.107; *t* = 3.261; *p* < 0.01), demonstrating that self-efficacy ultimately contributes to usage behavior through a multi-stage cognitive and attitudinal process.

Following structural model analysis, the study hypotheses were examined to determine whether they were supported. The path coefficients, *t*-values, and significance levels obtained from the bootstrapping analysis are presented in Table 7.

**Table 7:** Hypothesis testing results.

Hypothesis	Path	β	*t*-value	*p*-value	Result
H1	PEOU → PU	0.648^***^	10.845	< 0.001	Supported
H2	PU → ATU	0.638^***^	7.711	< 0.001	Supported
H3	PEOU → ATU	0.150^*^	2.164	0.031	Supported
H4	ATU → AU	0.399^***^	4.733	< 0.001	Supported
H5	PU → AU	0.195^*^	2.343	0.019	Supported
H5a	PU → ATU → AU	0.254^***^	3.837	< 0.001	Supported
H6	PEOU → AU	0.311^***^	5.380	< 0.001	Supported
H6a	PEOU → ATU → AU	0.060	1.882	0.060	Not Supported
H7	SE → PU	−0.116	1.410	0.158	Not Supported
H8	SE → PEOU	0.647^***^	12.183	< 0.001	Supported

^*^p < 0.05. ^**^p < 0.01. ^***^p < 0.001.

As shown in Table 7, all proposed hypotheses, except H6a and H7, were supported. Perceived ease of use had a significant and positive effect on perceived usefulness, supporting H1. Similarly, perceived usefulness has a strong and significant effect on attitude toward use, supporting hypothesis H2. Furthermore, the effect of perceived ease of use on attitude toward use was significant, supporting hypothesis H3. Examination of the effect of attitude toward use on actual usage behavior showed that attitude significantly and positively influenced actual use, supporting hypothesis H4. Furthermore, perceived usefulness was found to have a significant effect on actual use, supporting hypothesis H5. Furthermore, the effect of perceived usefulness on actual use of ChatGPT was mediated by teachers‘ attitudes toward ChatGPT, thereby supporting hypothesis H5a. Similarly, perceived ease of use had a direct and significant effect on actual use, supporting hypothesis H6. However, the indirect effect of perceived ease of use on actual usage behavior via teachers' attitudes was not statistically significant; therefore, hypothesis H8a was not supported.

Examination of the results for the self-efficacy variable revealed that self-efficacy had a strong and significant effect on perceived ease of use, supporting hypothesis H8. In contrast, the direct effect of self-efficacy on perceived usefulness was not statistically significant, and hypothesis H7 was not supported.

Overall, the findings confirm the basic assumptions of the TAM and show that perceived usefulness and perceived ease of use play a decisive role in explaining pre-service mathematics teachers‘ use of ChatGPT. Furthermore, including the self-efficacy variable in the model shows that individuals' perceptions of their competence in using technology indirectly influence technology acceptance, particularly via perceived ease of use.

These findings support the validity of the TAM in explaining the acceptance of AI-based tools in educational settings and provide important insights into the integration of GenAI tools, such as ChatGPT, into teaching processes.

## Discussion

The purpose of this study is to examine mathematics teachers' use of ChatGPT within the TAM framework and to highlight the role of pedagogical AI self-efficacy in this process. The research findings indicate that the fundamental mechanism underlying pre-service mathematics teachers' use of ChatGPT is largely explained by the relationships among the PEOU, PU, ATU, and AU variables. These results are consistent with theoretical assumptions that have long been accepted in the technology acceptance literature (Davis, 1989; Venkatesh and Davis, 2000; Venkatesh et al., 2003; King and He, 2006; Scherer et al., 2019; Ma et al., 2024).

The first key finding of the study is that PEOU strongly affects PU. This finding is also consistent with studies conducted in the Turkish context. For example, Teo et al. (2011) reported that perceived ease of use significantly predicts perceived usefulness among pre-service teachers in Türkiye. Similarly, recent research examining Turkish university students' acceptance of ChatGPT has shown that effort expectancy significantly predicts performance expectancy, reflecting the relationship between ease of use and perceived usefulness (Afacan Adanir, 2025). These findings support the stability of TAM relationships across both traditional and emerging technological contexts. This finding is consistent with both the classical TAM and its more recent extensions. According to TAM, when individuals perceive a technology as easy to use, they are more likely to evaluate it as useful (Davis, 1989; Venkatesh and Davis, 2000). Previous studies have consistently emphasized that perceived usefulness and perceived ease of use are fundamental determinants of technology acceptance (Venkatesh et al., 2003; King and He, 2006; Scherer et al., 2019; Ma et al., 2024). In line with this framework, the present study confirms that perceived usefulness is a strong predictor of attitudes toward ChatGPT, while perceived ease of use significantly influences both perceived usefulness and actual use.

Recent studies extending the TAM to emerging technologies such as artificial intelligence further support these findings. Research indicates that perceived usefulness and perceived ease of use remain significant predictors of attitudes and usage behavior in AI contexts (Chatterjee and Bhattacharjee, 2020; Dwivedi et al., 2023). These studies also emphasize that personal factors such as self-efficacy play an important role in shaping users' perceptions and technology acceptance. In line with this literature, the present findings confirm that TAM remains a robust framework for explaining the acceptance of AI-based tools such as ChatGPT.

Furthermore, extended TAM studies highlight the importance of self-efficacy as a key external variable influencing technology acceptance. Previous research has shown that individuals with higher self-efficacy are more likely to perceive technologies as easier to use, which indirectly enhances their acceptance (Teo, 2011; Scherer et al., 2019). Consistent with these findings, the results of the present study indicate that pedagogical AI self-efficacy plays a significant role in shaping perceived ease of use and indirectly contributes to the acceptance and use of ChatGPT.

In addition, recent studies examining the use of GenAI tools in education further support these relationships. Research shows that natural language-based AI systems such as ChatGPT enable users to access information and produce content without the need for complex technical procedures (Kasneci et al., 2023; Rudolph et al., 2023). Such systems can provide explanatory answers to users' questions, support problem-solving processes, and provide rapid feedback in learning processes (Rahman and Watanobe, 2023). It is also noted that GenAI tools may contribute to making learning processes more accessible by reducing users' cognitive load through natural language interaction (Dwivedi et al., 2023; Cotton et al., 2023). In this context, ChatGPT's user-friendly structure can be considered an important factor that increases perceived benefit, leading pre-service teachers to perceive the technology as more useful.

Research indicates that PU strongly influences attitudes toward the use of ChatGPT. The educational technology literature reveals that the perception that a technology will contribute to learning processes plays an important role in users developing positive attitudes toward that technology (Teo, 2011; Scherer et al., 2019). The ability of GenAI tools to provide explanatory answers to students' questions, to offer alternative solution strategies, and to explain complex concepts contributes to these tools being perceived as useful in teaching. Recent studies also show that ChatGPT and similar GenAI tools can support students' learning processes and offer new pedagogical opportunities in teaching activities (Kasneci et al., 2023; Cotton et al., 2023).

Another important finding of the study is that ATU is a significant predictor of ChatGPT's AU. This finding is consistent with the basic causal structure of the TAM model. The model suggests that individuals' positive evaluations of a technology can translate into technology usage behavior (Davis, 1989; Venkatesh et al., 2003; Ma et al., 2024). Studies in the field of educational technology also show that teachers' and pre-service teachers' perceptions and attitudes toward technology play a decisive role in technology integration processes (Ertmer and Ottenbreit-Leftwich, 2010; Scherer et al., 2019).

One important contribution of this study is its demonstration of the role of pedagogical AI self-efficacy in the technology acceptance process. The findings show that self-efficacy has a particularly strong effect on PEOU. This result is consistent with Bandura's (1997) self-efficacy theory. Self-efficacy refers to individuals' beliefs about their ability to perform a specific task, and it can influence their motivation to use new technologies. Pre-service teachers with high self-efficacy feel more confident about using new technologies, and this leads them to develop more positive perceptions in the technology learning process (Compeau and Higgins, 1995; Venkatesh and Davis, 1996; Teo et al., 2012).

Overall, the research findings show that the fundamental mechanism determining pre-service mathematics teachers' use of ChatGPT can be explained by the classic TAM progression: PEOU → PU → ATU → AU. However, pedagogical AI self-efficacy is an important external variable in this process, particularly through strengthening perceived ease of use.

### Practical implications

The findings of this study have important practical implications for teacher education programs and educational policies. The research results show that mathematics pre-service teachers' use of ChatGPT is largely shaped by PEOU, PU, and ATU. This highlights the need to increase application-based learning opportunities that support the pedagogical use of GenAI tools in teacher education programs.

In particular, offering practical training that allows pre-service teachers can use tools such as ChatGPT for teaching (for example, planning lessons, creating problems, and developing conceptual explanations) can foster positive perceptions of these technologies. Furthermore, the research findings indicate that SE plays a significant role in pre-service teachers' perceptions of technology use. Therefore, offering training on the pedagogical use of AI-supported teaching tools in teacher education programs can help pre-service teachers use these technologies more confidently and effectively.

### Theoretical contributions

This study makes important theoretical contributions to the literature on the adoption of GenAI tools in educational settings. The research findings show that TAM provides a valid theoretical framework for explaining the adoption of GenAI tools, such as ChatGPT. The relationships between the PEOU, PU, and ATU variables were found to meaningfully explain ChatGPT usage, and this result was consistent with TAM literature (Davis, 1989; Venkatesh et al., 2003; Scherer et al., 2019). Furthermore, the study expands the TAM literature by revealing the role of SE in the technology acceptance process.

## Limitations and future research

This study has some limitations. The research data were collected from mathematics pre-service teachers enrolled at specific universities. Future research involving larger samples and in-service mathematics teachers could provide more comprehensive information about how AI tools are perceived and used in actual classroom settings. One important limitation of this study is that both exploratory and confirmatory factor analyses were conducted on the same sample. This may increase the risk of overfitting and limit the generalizability of the factor structure. Therefore, future studies should validate the measurement model using independent samples. Furthermore, this study is based on a cross-sectional research design. Future longitudinal studies could examine how pre-service teachers' perceptions of AI tools and their usage change over time. In addition, including variables such as technology anxiety, digital literacy, AI trust, and ethical perceptions could contribute to developing more comprehensive models.

## Conclusion

GenAI tools, such as ChatGPT, are rapidly transforming teaching and learning environments. In this context, understanding how future teachers perceive and adopt these technologies is increasingly important. This study examined pre-service mathematics teachers' use of ChatGPT within the TAM framework and revealed the role of SE in this process. Research findings indicate that the use of ChatGPT is largely influenced by PEOU, PU, and ATU. Furthermore, SE plays a significant role in pre-service teachers' perceptions of technology use. Overall, the study presents important findings on factors influencing the adoption of GenAI tools in teacher education and highlights the need to integrate these technologies in a pedagogically grounded manner.

## Data Availability

The raw data supporting the conclusions of this article will be made available by the authors, without undue reservation.
